# Exploring the Pathological Role of Collagen in Paravertebral Muscle in the Progression of Idiopathic Scoliosis

**DOI:** 10.1155/2020/1527403

**Published:** 2020-08-03

**Authors:** Haidong Peng, Feng Jin, Depeng Meng, Jun Li, Shuhan Yu, Shen Zhang, Guigang Zeng

**Affiliations:** ^1^Department of Rehabilitation, Changzheng Hospital, Second Military Medical University, Shanghai, China; ^2^Shanghai University of Medicine & Health Sciences, Shanghai, China; ^3^Department of Orthopedics, Changzheng Hospital, Second Military Medical University, Shanghai, China

## Abstract

**Background:**

Paravertebral muscle (PVM) is considered as a contributing factor of idiopathic scoliosis (IS); collagen is crucial for maintaining the mechanical properties of PVM, but only a few researches have described this field. In this study, we observed the muscle stiffness of PVM and the curvature of the spine by adjusting the content of collagen in PVM of rats and explored the role of collagen in the progression of IS.

**Methods:**

32 female Sprague Dawley rats were randomly divided into four groups: neutralizing antibody (NA) group (group 1), normal control group (group 2), IS group (group 3), and IS with NA group (group 4). TGF-*β*1 NA was injected into PVM in group 1 and group 4, while Normal saline in group 2 and group 3. The Cobb angle and muscle stiffness were measured before and after injection; the rats were sacrificed at one week after injection, and performed histological, Western Blot, and qRT-PCR examinations.

**Results:**

X-rays showed that scoliosis occurred in group 1 and relieved in group 4. The stiffness of PVM was decreased significantly on the convex side in group 1, while on the concave side in group 4. The expression of TGF-*β*1 and COL1 on the concave side in IS rats (group 3) was significantly increased than that in normal rats (group 2), the concentration of COL1 and COL3 in group 3 was significantly higher than that in group 2, and the addition of TGF-*β*1 NA significantly downregulated COL1 and COL3 in group 1 and group 4. The concentration of COL1 in convex PVM was negatively related to Cobb angle in group 1 and group 2, and in concave PVM was positively related to Cobb angle in group 3 and group 4. However, no significant correlation was found between COL3 and Cobb angle in group 3 and group 4.

**Conclusions:**

Asymmetric biomechanical characteristics of PVM was an important etiological factor of IS, which was directly correlated with collagen, it could be adjusted by local intramuscular injecting of TGF-*β*1 NA, and finally had an effect on the shape of the spine.

## 1. Introduction

The idiopathic scoliosis (IS) was considered as a multifactorial disease, which was been proposed attributed to a number of factors, such as genetic, biochemical, and hormonal factors [1–4]. In addition, asymmetric loading on the spinal column may play roles in the onset and development of IS. The structures stabilizing the spine, including paravertebral muscle (PVM), have been suggested as implicated in the pathology of IS [5, 6].

Mechanical properties of muscle tissue are crucial in the biomechanical balance of the human body [7]. The biomechanical characteristics of PVM has been suggested as being scoliogenic (inducing scoliosis) or counteracting scoliosis in the initial development and maintenance of IS [8, 9]. Muscle tissue is a combination of muscle fibers and collagen fibers, muscle fibers are surrounded by connective tissue made of collagen fibers [10]. The active behavior of muscle usually refers to the contractile response of muscular fibers. Meanwhile, mainly responsible for the passive behavior of muscle is determined by collagen fibers [11, 12]. Previous studies have shown abnormal paraspinal electromyographic activity in PVM of IS patients [13, 14], which has been proved to be connected with the neuromuscular disorder [15]. However, the passive biomechanical characteristics related to collagen fibers in PVM are still under further research.

Despite the comparatively small amount of collagen, which accounts for only 1-10% of the muscle dry weight [16], it acts a central role in force transmission as it ensures the integrity and proper functioning of the entire muscle [17]. Fibrosis in the concave PVM was one of the noticeable pathological changes in IS patients [18]; It indicates that excessive collagen in concave PVM was an interesting target. We hypothesized that different collagen content in PVM resulted in asymmetry tension, and possibly related to the occurrence of IS. Transforming growth factor-*β*1 (TGF-*β*1) is the most important cytokine regulating collagen [19, 20]; in this study, TGF-*β*1 neutralizing antibody (NA) were injected into the right PVM of normal rats (to reduce collagen in right PVM) and left PVM of IS rats with right convex curve (to reduce the collagen in left PVM with collagen fibers hyperplasia). This could clarify whether the collagen in PVM would in fact induce the spinal deformity by asymmetric tension, thus having a scoliogenic effect, where changes of the spinal curve after reduction of collagen would happen.

## 2. Materials and Methods

### 2.1. Animals

**Figure 1:**
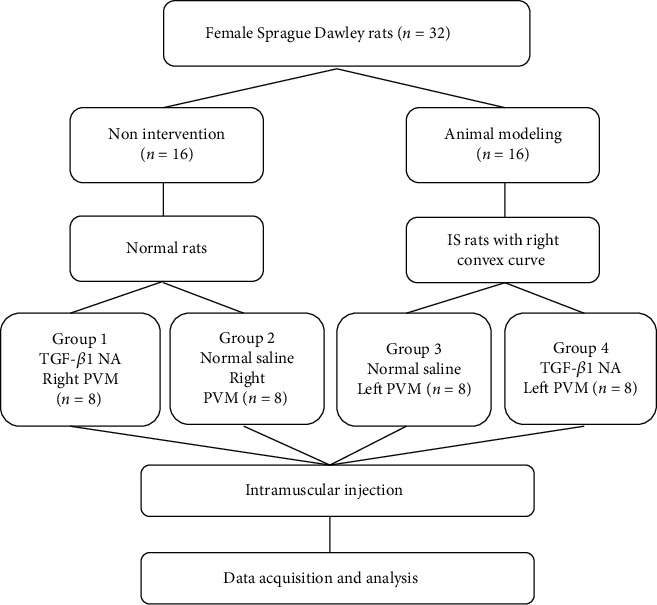
The study design.

32 3-month-old virgin female Sprague Dawley rats weighing 200–220 g were obtained from Shanghai Lab Animal Research Center. All experimental procedures conducted were approved by the Institutional animal care and use committee at the university of the second military medical university, Shanghai, China. In this study, rats were randomly divided into 4 groups of 8 animals each: TGF-*β*1 NA group (group 1), normal control group (group 2), IS group (group 3), and IS with TGF-*β*1 NA group (group 4), Figure 1. All groups were maintained on a laboratory rodent chow diet and housed in a laboratory at 22°C, humidity 40%, and light controlled (under a 12 h light and 12 h dark cycle).

### 2.2. Modeling Methods

**Figure 2:**
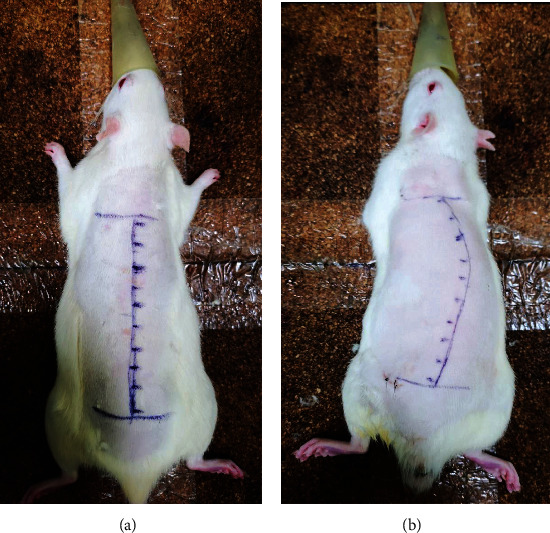
(a) Intramuscular injection points of PVM in group 1 and group 2. (b) Intramuscular injection points of PVM in group 3 and group 4.

Group 1: anesthesia was maintained by isoflurane inhalation, 10 points were evenly marked on the line connecting right scapula to the ipsilateral iliac wing (5 mm beside the spinous process) as the intramuscular injection points of PVM in a prone position, Figure 2(a). Each point was injected with 0.1 ml TGF-*β*1 NA (Mouse monoclonal antibody against rat TGF-*β*1; ab64715, Abcam, 0.5 mg/ml) diluted with normal saline (2.0 *μ*g/ml), once a week, three times in total.

Group 2: normal saline was injected into the right PVM. The injection site, dose, and frequency were the same as that of group 1, Figure 2(a).

Group 3: rats were anesthetized intraperitoneally with 2% barbitalum natricum (40 mg/kg) and underwent subcutaneous left scapula-to-ipsilateral iliac wing tethering procedure with nonabsorbable suture. The lower angle of the scapula and iliac wing were drawn together to 90% of the original distance, which made the spine convex toward the right side. Amputating the forelimbs at a high humeral level and the tails at the root to create the bipedal rats. After removing the forelimbs and tail, the bipedal rats were housed in special high cages with raised food and water to ensure that they maintained a standing posture most of the time; both food and water were gradually elevated in the cage as the bipedal rats grew [21, 22]. The sutures were cut to complete the modeling at postoperative 8 weeks. One week after the completion of modeling, the Cobb angle was greater than 10°, indicating that the modeling was successful. Normal saline was injected into the left PVM; the injection site, dose, and frequency were the same as that of group 1, Figure 2(b).

Group 4: one week after IS models were made successfully, TGF-*β*1 NA was injected into the PVM of the left side. The injection site, dose, and frequency were the same as that of group 3, Figure 2(b).

To avoid the errors by employing different operators, all experimental procedures were accomplished by the same experimenter and assistant.

### 2.3. Imaging Examination

X-ray of full-length radiographs was taken by In-Vivo Imaging System FX Pro (Kodak, USA) to evaluate the Cobb angle before injection and one week after injection. Rats in all groups were anesthetized by isoflurane and involved positioning them as straight as possible without any applied traction. Cobb angle at the primary curve in the coronal planes were measured by Digimizer software. The location of the upper or lower vertebra was together identified by two experienced evaluators of radiology. If there were different views, the average was selected.

### 2.4. Muscle Stiffness

**Figure 3:**
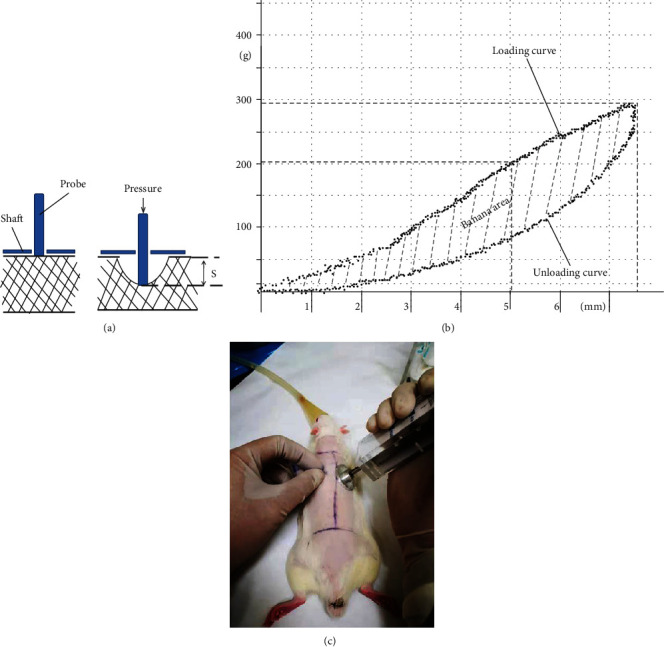
(a) Schematic diagram of muscle stiffness test principle. (b) The interface of data acquisition and data displaying, *x*-axis shows the distance travelled, whereas the *y*-axis to show the vertical downward compressive force in each position. (c) The operation method of M_tone Soft Tissue Elasticity Meter.

Muscle stiffness is defined as the degree of deformity of the muscle to given a pressure [23, 24]. Applying the same pressure, the higher degree of muscle stiffness is, the smaller is the tissue displacement. It has been proved to be a reliable noninvasive examination by many researches that the muscle stiffness can be measured by the force-displacement curve [25] In this study, M_tone Soft Tissue Elasticity Meter (JZL-111, Tianjin Mingtong Century Technology Co., Ltd.) was applied to examine the muscle stiffness of bilateral PVM after radiological examination. The M_tone Soft Tissue Elasticity Meter consists of a metal probe, surrounded by a metal shaft. When the tester applies vertical downward compressive force, the shaft remains stationary on the surface of the skin while the probe compresses the tissue to produce deformation, Figure 3(a). In the process of detection, all data were registered and stored in a computer attached to the device, and two real-time force-displacement curves, loading curve, and unloading curve showed on computer based on these data simultaneously. In this study, muscle stiffness of PVM was reflected by the displacement at 200 g pressure (D_200_) on the loading curve. The proportion of “banana” area (PBA) between the two curves in the total area under the loading curve, which is correlated with the degree of muscle spasm or tissue stiffness, the higher PBA was, the less muscle spasm it was [26], Figure 3(b).

During the measurement, rats were anesthetized by inhalation of isoflurane with lying prone; reference test points were marked on both sides at the apex of curve 5 mm beside the spinous process, Figure 3(c).

### 2.5. Tissue Specimens

PVM was harvested from the right side (groups 1 and 2) or left side (groups 3 and 4). Muscle samples were placed in separate sterile tubes and immediately stored in liquid nitrogen. Frozen samples were stored at -80°C until analysis.

### 2.6. Histological Evaluation of Fibrosis

The muscle samples were fixed in 10% formalin, embedded in paraffin blocks, and cut into 5 *μ*m thickness slices. Masson trichrome staining was carried out to estimate the severity of fibrosis in PVM as follows [27]: Cross sections were deparaffinized, rehydrated, washed in running water for 2 min, immersed in Weigert's iron hematoxylin for 15 min, and rinsed in distilled water, then washed in running water for 5 min. Then, sections were stained with 1% Biebrich scarlet-acid fuschin for 15 min and washed in distilled water for 5 min. After differentiation in 2.5% phosphomolybdic-phosphotungstic acid solution for 15 min, sections were transferred directly into 2.5% aniline blue solution for 12 min. Muscle sections were then differentiated in 1% acetic acid solution for 3 min, dehydrated in 95% and 100% ethanol, then cleared in xylene. Muscle fibers were stained red and collagen fibers blue in Masson staining. The ratio of the fibrotic area to the total cross-sectional area was calculated to estimate the severity of fibrosis in PVM.

### 2.7. Western Blot

The protein samples were carefully separated and washed with sterile phosphate-buffered saline. Then, samples were stored in a saline-soaked gauze at −20°C until analysis. Separated proteins were blot transferred onto a nitrocellulose membrane. After blocking with 0.1% Tween 20 and 5% nonfat milk in tris-buffered saline at room temperature for 1 h, the membrane was incubated overnight at 4°C in the following primary antibody: Anti-TGF beta 1 (Abcam,ab92486), Anti-Collagen I Rabbit pAb (GB11022-2), Anti-Collagen III Rabbit pAb (GB11023), and *β*-actin (Santa Cruz, CA, USA) as an internal control. The intensity of protein bands was measured, and the protein expression levels were normalized to *β*-actin concentration.

### 2.8. qRT-PCR

To assess the concentration of COL1 and COL3, the muscles were homogenized in Trizol reagent using TissueLyser (TissueLyser II, QIAGEN Inc., Germany). RNA was isolated according to the manufacturer's guidelines. The RNA was reverse transcribed to cDNA and diluted 1 : 10. Quantitative Real-Time Polymerase Chain Reaction (qRT-PCR) analysis of COL1 and COL3 using the TaqMan™ Gene Expression Assays (Thermo Fisher Scientific, Canada) Table 1.

The relative amount of mRNA in the muscle compared with the Gapdh genes was computed according to the comparative Ct method. We computed the relative gene expression for each of the genes in tissue from the instrumented level by subtracting the Ct value of an endogenous control gene from the Ct value of the experimental gene, yielding the *△*Ct value. Then, we further normalized *△*Ct values of the instrumented samples to the *△*Ct values of the internal control discs, yielding the *△△*Ct value. Finally, assuming that each strand of cDNA in the sample is copied exactly once per PCR cycle (giving an exponential relation between the cycle number and quantity of cDNA), we used the comparative Ct value method (2^-*△△*Ct^) in statistical analyses.

### 2.9. Statistical Analysis

All statistical analyses were performed using the SPSS 18.0 (IBM Corp, Armonk, NY). Paired *T* test was used to compare the difference of muscle stiffness in bilateral PVM. Independent-Sample *T* test was used to compare the Cobb angle, protein expression, mRNA concentration, and fibrotic area between groups. Finally, Pearson correlation analysis was conducted to analyze the correlations between parameters. All analyses values were expressed as mean ± standard deviation (SD), and the significance level of the tests was set at 0.05.

## 3. Results

### 3.1. Imaging Examination

**Figure 4:**
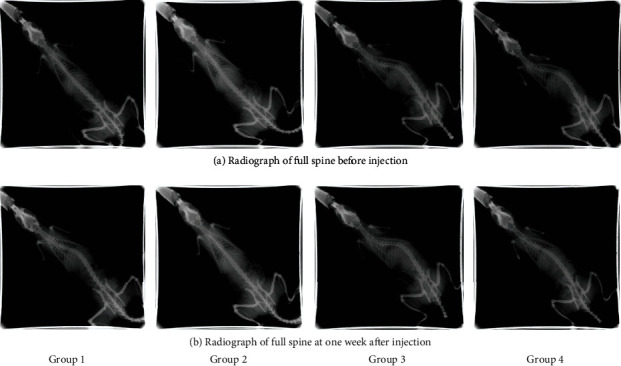
Comparison of radiograph of full spine between groups.

**Figure 5:**
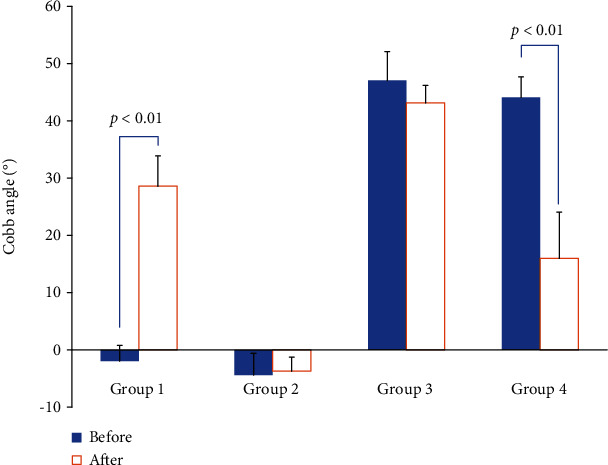
The Cobb angle between groups.

One week after injection, X-rays showed that scoliosis occurred in group 1, conversely, relieved in group 4. The Cobb angle in group 1 (ranged from 23.6° to 42.3°) was gradually increased compared with that in group 2 (ranged from -6.1° to 6.3°). In group 4, the Cobb angle (ranged from 8.4° to 29.5°) was decreased significantly than that in group 3 (ranged from 41.7° to 54.7°). Anteroposterior views of spinal radiographs are shown in Figure 4, and overall scoliosis rates recorded are shown in Figure 5.

### 3.2. Muscle Stiffness

**Figure 6:**
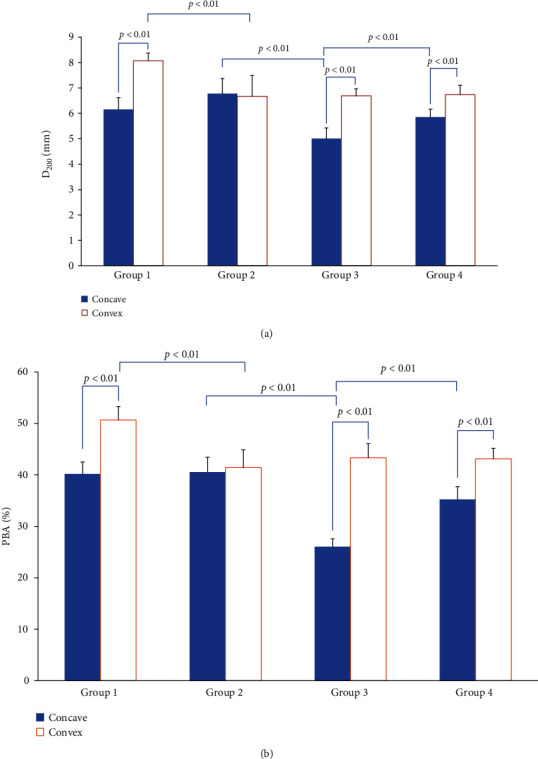
(a) Comparison of bilateral D_200_ between groups. (b) Comparison of bilateral PBA between groups.

The imbalance muscle stiffness of bilateral PVM widely existed in group 1, group 3, and group 4, in which there were different degrees of scoliotic curve. The stiffness and spasticity of PVM on the concave side in IS rats (group 3) was significantly different from that of normal rats (group 2). After injection, the D_200_ and PBA was increased significantly on the convex side in group 1, while was decreased significantly on the concave side in group 4. Figures 6(a) and 6(b) list the results of the M_tone examination in the bilateral comparison of the scoliosis curve.

### 3.3. Western Blot

**Figure 7:**
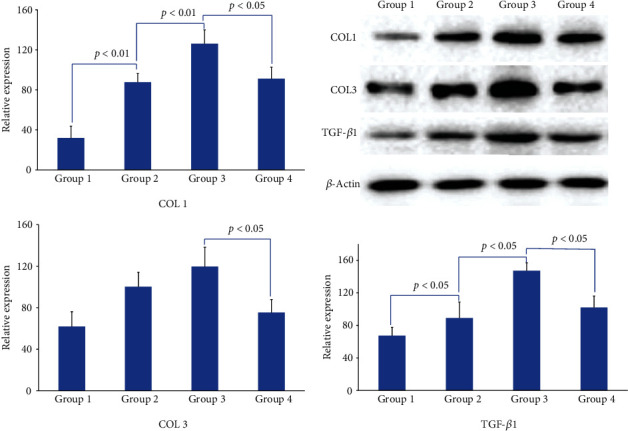
Quantification of COL1, COL3, and TGF-*β*1 expression in PVM between groups.

The protein expression of TGF-*β*1 and COL1 on the concave side in IS rats (group 3) was significantly increased than that in normal rats (group 2, *p* < 0.01), COL3 in IS rats also increased, but there was no statistical significance (*p* = 0.1332). Addition of TGF-*β*1 NA resulted in a significant decrease of TGF-*β*1 and COL1 in group 1 (vs. group 2, *p* < 0.05) and group 4 (vs. group 3, *p* < 0.05), COL3 decreased in group 1, but there was no statistical significance (vs. group 2, *p* = 0.3360), Figure 7.

### 3.4. qRT-PCR

**Figure 8:**
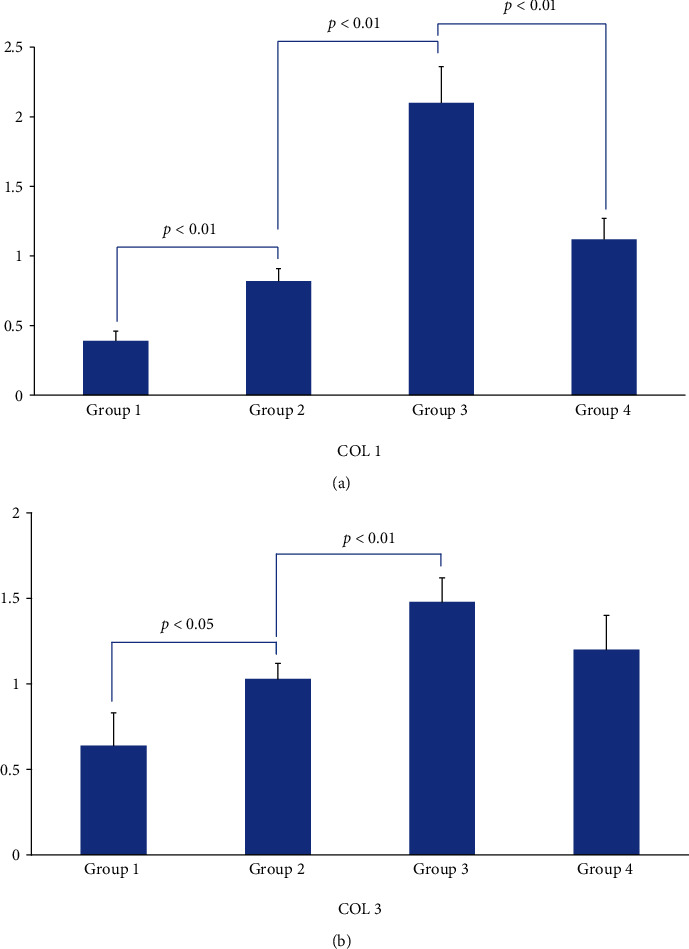
(a) The concentration COL1 in PVM. (b) The concentration COL3 in PVM.

The concentration of COL1 and COL3 in group 3 was significantly higher than that in group 2 (*p* < 0.01). The addition of TGF-*β*1 NA significantly downregulated the concentration of COL1 in group 1 and group 4 (*p* < 0.01), but the decrease of COL3 in group 4 has no statistical significance (*p* = 0.1345), Figures 8(a) and 8(b).

### 3.5. Fibrotic Area Assessment

**Figure 9:**
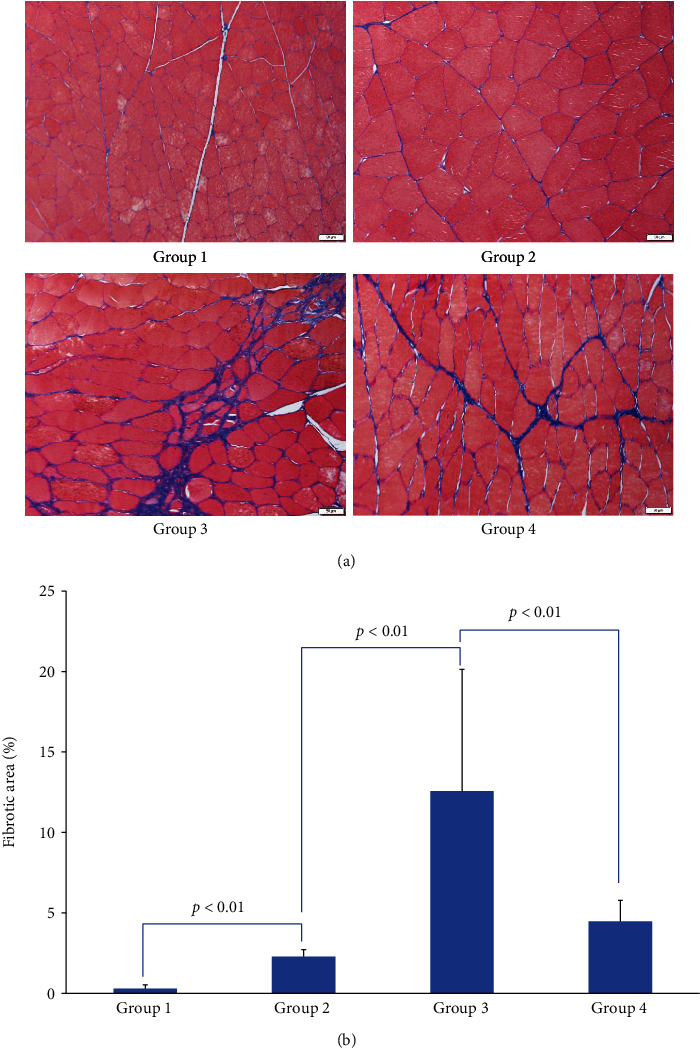
(a) Muscle fibrosis in PVM in different groups. Masson trichrome staining was used to determine the total amount of collagen in the muscle tissue. Collagen was stained blue; myofibers were stained red (Masson staining, ×200, Scale bar on pictures is 50 *μ*m). (b) Quantification of the proportion of fibrotic area to the total cross-sectional area.

The proportion of the fibrotic area in concave PVM in group 3 was significantly higher than group 2 (*p* < 0.01). The positive fibrotic areas were significantly less prevalent in the TGF-*β*1 NA groups (group 1 vs. group 2, *p* < 0.01; group 3 vs. group 4, *p* < 0.01), Figures 9(a) and 9(b).

### 3.6. Correlational Analyses

The muscle stiffness of PVM was closely related to the Cobb angle; there was a significant negative correlation between the difference value of muscle stiffness on bilateral PVM (*△D*_200_ or *△*PBA) and Cobb angle. The concentration of COL1 in convex PVM was negatively related to Cobb angle in group 1 and group 2 and was positively related to Cobb angle in group 3 and group 4 in concave PVM. However, no significant correlation was found between COL3 and Cobb angle in group 3 and group 4, Table 2.

## 4. Discussion

### 4.1. PVM and IS

The etiology of IS is linked to many factors; studies support that spinal deformity may be associated with PVM atrophy. PVM lesion is more often observed on the concave side among patients with IS, which is positively connected with the progression of IS [6]. The muscle thickness was significantly greater on the concave side of the curve compared with the control's dominant side [28]. The muscle volume and fatty infiltration in deep PVM of adolescent IS patients were imbalance [29]. The type sizes and distributions of muscle fiber were different in the concave and convex sides in IS [30]. Magnetic resonance imaging and histology showed that muscle degeneration was more common on the concave side in patients with IS [31]. The presence of PVM disorder has been suggested as a plausible cause for scoliosis [32].

PVM is vital for the stability and functional movement of the spinal column; tension imbalance is a key factor in the initiating or maintaining scoliotic curvature by muscle contraction [33]. The stiffness of concave PVM was greater than that on the convex side in IS patients. The asymmetric biomechanical characteristics of PVM are related to the severity of scoliosis [8]. We found that the asymmetric muscle stiffness of PVM was well correlated with the Cobb angle that implied PVM played a scoliogenic role by performing a lateral pull or contraction. As we supposed earlier, if the excessive muscle pull by contraction was reduced in the concave side with the subsequent effect in the reduction of Cobb angle in IS rats. Conversely, the unilateral insufficient muscle traction leads to increased Cobb angle, thus creating a scoliosis in normal rats.

### 4.2. Collagen and the Mechanical Properties of PVM

Mechanical properties of skeletal muscle are crucial in spinal stability, which is determined by its composition. Skeletal muscle is a combination of muscle fibers and connective tissue including collagen fibers [7]. Collagen fibers maintain the integrity of skeletal muscle by binding muscle fibers together and allow the transmission of muscular forces [34]. Skeletal muscle was considered as a three-dimensional network structure of muscle fibers, collagen fibers embedded in an isotropic matrix [35]. From the internal to the external level, the muscle fibers are organized histologically by the surrounding connective tissues of three different properties made of collagen fibers: the endomysium surrounds individual fibers; the perimysium binds muscle fibers into fascicles and the epimysium surrounds the whole muscle [36, 37].

The active response of skeletal muscle is responsible for the contractile behavior, usually refers to the response of the muscular fiber [11, 12]. Meanwhile, the passive mechanical properties of skeletal muscle clearly depend on the size, orientation, and organizational arrangement of collagen fibers, particularly in the perimysium [38]. These pathologic changes of collagen hyperplasia induce muscle contracture and finally increased passive stiffness [39, 40]. Muscle passive stiffness can contribute to the loss of range of motion about the spine, leading to further loss of function. Muscle stiffness is directly correlated with total collagen content [41, 42], which is consistent in our experimental results. Furthermore, we found that the muscle stiffness of PVM was positively related to COL1, however, was not correlated with COL3. This discrepancy could be explained by the proportion of different collagen types. In skeletal muscle, COL1 is predominant, whereas COL3 accounts approximately for 10% of total collagen [43]. COL1 resists tension and COL3 provides structural stability [44], difference of arrangement, and size of collagen fibers resulted in COL1 is more resistant than COL3 [45]. In our study, muscle stiffness and Cobb angle were positively related to COL1 in PVM, while were not correlated with COL3 in group 3 and group 4. This result suggests that the abnormality of COL1 has a closer relation to the progression of IS.

### 4.3. Fibrosis of PVM and IS

PVM degeneration presents very frequently on the concave side of the scoliotic curve, which was morphologically abnormal in patients with IS [6]. Fibrosis, an excessive accumulation of collagen, in skeletal muscle impairs its function [40, 46], is a cause of increased stiffness of skeletal muscle. TGF-*β*1 is considered critical regulators of physiological fibrogenesis and pathological fibrosis in kidneys, liver, lungs, and skeletal muscles [47]. In previous studies, tissue samples collected from IS patients indicated that TGF-*β*1 in PVM, vertebral cartilaginous endplates, or articular cartilages of the scoliosis apex was significantly higher on the concavity [48]; the fibrosis of muscular samples was more serious on the concave side [18]. With the deteriorated muscular fibrosis on the concavity, the prominent difference of TGF-*β*1was observed on both sides showed that TGF-*β*1 was involved either as an etiological factor or a secondary change in the scoliosis progression [49].

To our knowledge, this was the first study, in which PVM was influenced directly by intramuscular injection of TGF-*β*1 NA in order to explore the role of collagen in the progression of IS. Collagen in our hypothesis was being an important step for the further exploration and understanding of the etiology of IS. TGF-*β*1 NA were injected into the right PVM of normal rats to reduce the content of collagen, resulting in a drop of muscle stiffness on the right side, and produce a right convex curve. Similarly, the injection of TGF-*β*1 NA into the concavity of IS rat could alleviate the degree of PVM stiffness and reduce the Cobb angle. We found that the content of collagen was closely related to muscle stiffness. By reducing the content of collagen, we could adjust the asymmetric biomechanical characteristics of bilateral PVM and finally had an influence on the formation of the spinal column.

## 5. Conclusions

In conclusion, asymmetric biomechanical characteristics of PVM is an important etiological factor of IS, which is directly correlated with collagen. By local intramuscular injecting of TGF-*β*1 NA to reduce the content of collagen in PVM, the asymmetric tension of PVM can be adjusted; finally, it has an effect on the shape of the spine. The identification of significant asymmetry in the content of collagen between the concave and convex side of the scoliotic curve could suggest a new perspective of progression risk and aid in developing novel therapeutics to combat structural changes of the spine.

## 6. Limitations

However, in this study, we detect an antifibrosis treatment for short-term effects and only focused on the quantity of collagen. It is obvious that in such multiple factorial disease as IS, one factor cannot be the only participant responsible for spine curvature dysfunction, precise mechanisms of collagen still remain elusive. Thus, further tests are necessary to determine the ratio of COL1/COL3, which is important and might influence the biomechanical property of PVM, or the dose-effect relationship of TGF-*β*1 NA. These further studies will bring us additional to understand the roles of collagen in the progression of scoliosis that may allow us to effectively improve spinal deformity.

In addition, in groups 3 and 4, we adopted the approach of bipedal unilateral tethering to establish scoliosis model, which was a mature and standard modeling method widely used in studies of scoliosis, while adopted quadrupedal rats in groups 1 and 2. Due to rodents exhibit a preference for erect walking and phylogenetically, they are still quadrupeds, some studies suggested that the upright posture may not be the difference of some previously reported between bipedal and quadrupedal rats, bipedal rat spends no more time in an upright position compared with quadrupedal rats [50, 51]. The gravity seemed to have been a relatively minor factor and would not lead to significant error of the experimental results. However, the consistency between groups still need improvement in our following study, and how gravity and collagen contribute to scoliosis deserves further study.

## Figures and Tables

**Table 1:** Primers sequences for qRT-PCR.

Gens	Direction	Primers (5′-3′)
*Gapdh*	Forward	TCTCTGCTCCTCCCTGTTC
	Reverse	ACACCGACCTTCACCATCT
*COL1*	Forward	GGTTGCAGCCTTGGTTAG
	Reverse	TGAGCCAGCAGATTGAGAA
*COL3*	Forward	GGTTTGGAGAATCTATGAATGGTGG
	Reverse	GCTGGAAAGAAGTCTGAGGAAGG

**Table 2:** Linear regression analysis of muscle stiffness, collagen with Cobb angle.

	Group	*n*	Coefficient^∗^	*R* ^2^ linear	*p* value
Muscle stiffness					
*△*D_200_	Groups 1, 2, 3, 4	32	-0.841	0.708	<0.001
*△*PBA	Groups 1, 2, 3, 4	32	-0.915	0.839	<0.001
Collagen content					
COL1	Groups 1, 2	16	-0.900	0.810	<0.001
	Groups 3, 4	16	0.923	0.852	<0.001
COL3	Groups 1, 2	16	-0.539	0.291	0.031
	Groups 3, 4	16	0.448	0.201	0.082

^∗^Pearson correlation coefficient.

## Data Availability

The data used to support the findings of this study are available from the corresponding author upon request.

## Abbreviations

IS: Idiopathic scoliosis
PVM: Paravertebral muscles
TGF: Transforming growth factor
NA: Neutralizing antibody
COL: Collagen
D_200_: Displacement at 200 g pressure
PBA: Proportion of “banana” area
ECM: Extracellular matrix.
